# Large-strain anisotropic behavior of meat during cooking: A finite element study^☆^

**DOI:** 10.1016/j.crfs.2025.101230

**Published:** 2025-10-28

**Authors:** Jorge Grasa, Luciano Teresi, Ruud van der Sman

**Affiliations:** a Aragón Institute of Engineering Research (I3A), Universidad de Zaragoza, Zaragoza, Spain; b Centro de Investigación Biomecánica en Red en Bioingeniería, Biomateriales y Nanomedicina (CIBER-BBN), Zaragoza, Spain; c Roma Tre University, Italy; d Wageningen Food & Biobased Research, The Netherlands; e Food Process Engineering, Wageningen University & Research, The Netherlands

**Keywords:** Meat cooking, Anisotropic large deformations, Multiphysics, Finite elements

## Abstract

This paper investigates the role of anisotropy in the deformation of meat during cooking, and how it influences transport properties such as diffusion and thermal conductivity. To address this multiphysics problem a Finite Element model was developed, coupling mass transfer of water with thermal energy transport and large-strain solid mechanics. To account for fiber directionality, an anisotropic constitutive model with swelling-driven deformation is implemented. Particular emphasis is given to the definition of the reference configuration. Numerical simulations reveal that fiber orientation significantly influences the local stretch and overall shape evolution during pan frying. The magnitude and anisotropy of the deformation are sensitive to the swelling parameters and the fiber stiffness. These findings offer insights into the physical origin of cooking-induced texture transformations in meat and provide a framework for modeling fibrous biological materials undergoing thermal and fluid-driven processes.

## Highlights

• Large deformation mechanics is coupled with heat and mass transfer to model meat pan frying.
• Mechanical behavior is modeled as transversely isotropic to reflect fiber alignment.
• Heat and mass transfer parameters are direction-dependent, varying along and across the fibers.
• Fiber orientation strongly governs local deformation and macroscopic shape evolution.

## Graphical abstract

**Figure:**
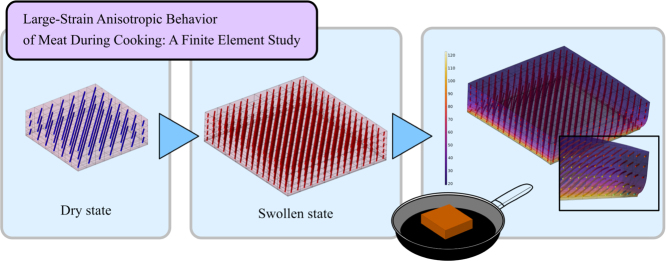

## 1 Introduction

Understanding the mechanics and thermomechanical behavior of animal meat during cooking has become critical for developing plant-based and cultured alternatives that accurately replicate traditional meat’s sensory experience. Key focus areas include how muscle fiber contraction and collagen denaturation govern texture evolution, moisture retention, and structural changes during heating (Tahir and Floreani, 2022, St. Pierre et al., 2023, Kolel et al., 2023, Avegnon et al., 2024, St. Pierre et al., 2024, Chen et al., 2024). The challenge is mimicking meat’s hierarchical microstructure of aligned myofibers bundled with collagenous connective tissue (endomysium, perimysium, epimysium) across multiple scales, from single fibers to whole muscles connected to tendons since this structure critically determines texture and thermal response.

The cooking of meat is intriguing from a physics perspective, as it significantly affects both the mechanical properties and juiciness of the meat van der Sman, 2007, van der Sman, 2013, Spyrou, 2020, Moya et al., 2021. Through cooking procedures, one can steer the texture of animal meat, such as beef steak, from rare to medium-rare and well-done (Moya et al., 2021). Previous work has explored the modeling of muscle meat cooking such as beef or chicken fillets using Flory–Rehner theory to describe moisture expulsion during heating. However, earlier models (van der Sman, 2007, van der Sman, 2013) neglected the mechanical aspects of the problem, particularly meat shrinkage. More recently, Flory–Rehner theory has also been applied to describe juiciness in plant-protein-based meat alternatives (Cornet et al., 2021, Cornet et al., 2020b, Cornet et al., 2020a).

Both shrinkage and water loss are consequences of muscle protein denaturation (Kovácsné Oroszvári et al., 2006). Fiber anisotropy influences material properties across all physical processes, including the stiffness, thermal conductivity, and moisture diffusion or permeability. For the mechanical response, the modeling approach builds on established biomechanical frameworks in which muscle tissue is treated as a transversely isotropic material (Nardinocchi and Teresi, 2013, Alharbi et al., 2022). The effects of heating on muscle tissue, however, have received limited attention, despite their relevance to medical applications such as thermal ablation (Molinari et al., 2022). The only existing study that incorporates protein denaturation (Park et al., 2018) links the resulting shrinkage to thermoelastic (plastic) deformation, rather than to changes in thermodynamic interactions as represented by a temperature-dependent Flory–Huggins parameter.

During meat cooking, shrinkage has been found to be particularly pronounced along the fiber direction (van der Sman, 2013), but it is preceded by shrinkage in the transverse direction at lower temperatures (Vaskoska et al., 2020). The relative magnitudes of longitudinal and transversal shrinkage depend on muscle type, and their particular hierarchical structure of muscle tissue, where myofibers and connective tissue have different denaturation responses (Kong et al., 2008, Purslow et al., 2016). In principle, the mechanical behavior of real muscle meat is more intricate than what is captured by a transversely isotropic model. Several advanced mechanical models have been developed to account for this hierarchical organization and the role of connective tissue (Sahani et al., 2024, Almonacid et al., 2024). From a numerical standpoint, coupling a transversely isotropic mechanical model with heat and mass transfer is already a considerable and innovative challenge.

Pan frying is a widely used technique for cooking meat, valued for its ability to create desirable texture and flavor. Recent advances in computational modeling have enabled detailed simulations of the coupled thermo-mechanical processes involved in double-sided cooking of beef steak (Moya et al., 2021) and burgers (Hernández-Alhambra et al., 2024). These models simultaneously resolve conductive heat transfer between the pan and meat, moisture diffusion driven by pressure gradients resulting from protein denaturation, and volumetric deformation due to myofiber shrinkage. They provide physics-based guidance for achieving desired doneness while minimizing energy use and establishing computational tools as viable kitchen assistants. However, the mechanical behavior of meat is treated as isotropic in these models, which may be justified for minced meat such as burgers, but not for whole-muscle cuts like steaks, where anisotropy plays a significant role

This paper presents a model that represents a significant advancement toward capturing the actual mechanics of meat cooking. The model accounts for the anisotropic, large deformation of muscle meat, accompanied by the expulsion of water. From a multiphysics simulation point of view the introduction of anisotropy is challenging. Consequently, a simple transverse-isotropic strain energy function is adopted to implement anisotropy in the large deformation. While realistic anisotropic deformation would require a more complex orthotropic model capable of accounting for the effects of enveloping connective tissue (epimysium), such an approach is considered beyond the scope of this work. The focus remains on the implementation of solvable multiphysics models with moderate anisotropy in deformation, permeability, and thermal conductivity.

## 2 Mathematical framework

### 2.1 State variables

**Fig. 1:**
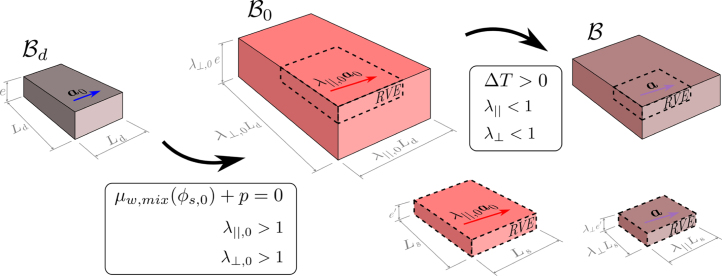
Initial free-swollen state determination in a 3D domain and subsequent drying process. Left: Chemical equilibrium leads to stretches greater than unity. Right: A temperature increase results in shortening both along and across the fiber direction. The Reference Volume Element (RVE) illustrates the change in dimensions and fiber orientation vector length.

To simulate the cooking process, the material is first considered in its completely dry state $B_{d}$ and allowed to swell under isothermal conditions, with the temperature held constant at its initial value, until it reaches the free-swollen state $B_{0}$, which serves as the initial configuration for the subsequent heating stage. In this phase, the temperature within the material increases due to external heating, triggering protein denaturation and structural contraction. These processes reduce the material’s ability to retain water, leading to moisture expulsion as cooking progresses. Fig. 1 illustrates these processes, in which the actual state B is reached after the temperature increase. A detailed description of the steps, along with the resulting deformations and dimensional changes in this idealized geometry, is provided in the following sections

The state variables describing the condition of the meat at each instant in time, including the reactive pressure, are:

(1) $$\begin{array}{cc} u:B_{0}\times T\to V & \text{displacement from}B_{0}\text{,}\left[u\right]=\text{m;}\\ c_{d}:B_{0}\times T\to R & \text{water concentration in}B\text{,}\left[c_{d}\right]=\text{mol/m}{}^{3}\text{.}\\ p:B_{0}\times T\to R & \text{reactive pressure in}B\text{,}\left[p\right]=\text{Pa;}\\ T:B\times T\to R & \text{temperature in}B\text{,}\left[T\right]=\text{K;} \end{array}$$

Four primary fields defined over the body B and the time domain T are considered. The displacement field u(X,t) describes the motion of material points $X\in B_{0}$ from their reference configuration, expressed in meters to their current position x∈B as x=X+u(X,t)=f(X,t), where f is the motion; $B=f\left(B_{0},t\right)$ is called current configuration. As defined in Eq. (1), square brackets denote the units of a quantity. For instance, [u]=m indicates that the displacement u is measured in meters.

The water concentration $c_{d}\left(X,t\right)$ quantifies the amount of water per unit volume within the body, in units of moles per unit of reference volume (m${}^{3}$). The reactive pressure p(X,t) represents the hydrostatic pressure generated by chemical and mechanical processes, measured in pascals, and is defined in the initial configuration $B_{0}$. Finally, the temperature field T(X,t) specifies the thermal state of the body in kelvin.

To ensure notational consistency and facilitate subsequent mathematical developments, the gradient operator with respect to material coordinates X (reference configuration) is denoted by ∇, while the gradient with respect to spatial coordinates x (current configuration) is denoted by $\nabla_{\;x}$. This convention will be maintained consistently throughout this work.

### 2.2 Constitutive relationships

Based on the classical Flory-Rehner theory, the anisotropic mechanical behavior of the meat material can be modeled using a free energy density function (per unit reference volume). This function includes two distinct contributions along with a term that ensures the volumetric incompressibility arising from the interaction between the solid protein network and the water solvent.

(2) $$\Psi =\Psi_{\text{mix}}\left(\phi_{s},T\right)+\Psi_{\text{mech}}\left(J,I_{1},I_{4}\right)-p\left(J-\left(1+\Omega c_{d}\right)\right)$$

The mixing contribution $\Psi_{\text{mix}}$, written as a function of the volume fraction of proteins ($\phi_{s}$) and the temperature (T), describes the thermodynamic interaction between moisture and proteins, which will follow the Flory–Huggins theory (van der Sman, 2012, van der Sman, 2013).

(3) $$\Psi_{\text{mix}}=\frac{RT}{\Omega }\frac{1}{\phi_{s}}\;\left(\left(1-\phi_{s}\right)\ln\left(1-\phi_{s}\right)+\chi \phi_{s}\left(1-\phi_{s}\right)\right)$$

where R is the gas constant and Ω is the molar volume of water. Finally χ, the Flory–Huggins interaction parameter that is composition dependent (van der Sman and Meinders, 2011, van der Sman, 2013) can be written as:

(4) $$\chi \left(T\right)=\chi_{0}+\left(\chi_{1}-\chi_{0}\right)\phi_{s}^{2}$$

For meat, $\chi_{1}$ is temperature dependent to account for protein denaturation (van der Sman, 2013) and an explicit expression will be shown later in the Results section.

The mechanical contribution $\Psi_{\text{mech}}$ is formulated in terms of the Jacobian J=det(F), where F=I+∇u is the deformation gradient, the first invariant ($I_{1}$) of the right Cauchy–Green deformation tensor $C=F^{T}F$, and a pseudoinvariant $I_{4}=a_{0}\cdot \left(Ca_{0}\right)$ that accounts for anisotropy, with $a_{0}$ the fiber reference-direction. For a material with one family of fibers, this work adopts the following expression for $\Psi_{\text{mech}}$ (Liu et al., 2015):

(5) $$\Psi_{\text{mech}}=\frac{1}{2}G_{0}\left(I_{1}-3-2\ln J\right)+\frac{1}{2}G_{0}\gamma {\left(I_{4}-1\right)}^{2}$$

where $G_{0}$ is the shear modulus, and γ the reinforcement factor.

To ensure incompressibility, the following relationship between protein volume fraction and water concentration is enforced by the Lagrangian multiplier p.

(6) $$J=\frac{1}{\phi_{s}}=1+\Omega c_{d}$$

For the derivation of appropriate constitutive relations for stresses, chemical potential, and water flux, the local dissipation inequality, considered in the reference configuration at a specific temperature (Chester and Anand, 2010, Duda et al., 2010, Curatolo et al., 2018), can be written using a dot over a quantity to denote its time derivative, as follows:

(7) $$\overset{˙}{\Psi }\leq \mu {\overset{˙}{c}}_{d}+P:\overset{˙}{F}-J_{w}\cdot \nabla \mu_{w}-\left(J-\left(1+\Omega c_{d}\right)\right)\overset{˙}{p}$$

where P is the first Piola–Kirchhoff stress, $\mu_{w}$ is the chemical potential of water, and $J_{w}$ is the water molar-flux, $\left[J_{w}\right]=$mol/(m${}^{2}$ s).

This imbalance expresses that the time rate of the free energy within any part of the domain must be less than or equal to the sum of chemical and mechanical power inputs, minus the energy dissipated by fluid transport, and corrected by the volumetric constraint imposed by incompressibility. From (2), it follows the free-energy rate:

(8) $$\overset{˙}{\Psi }=\left(\frac{\partial \Psi_{\text{mix}}}{\partial c_{d}}+p\Omega \right){\overset{˙}{c}}_{d}+\left(\frac{\partial \Psi_{\text{mech}}}{\partial F}-pJF^{-T}\right)\cdot \overset{˙}{F}-\left(J-\left(1+\Omega c_{d}\right)\right)\overset{˙}{p}$$

The inequality (7) is to hold for all values of $c_{d}$, F, μ, and the incompressible constraint, therefore, it can be identified that:

(9) $$\mu_{w}=\frac{\partial \Psi_{\text{mix}}}{\partial c_{d}}+p\Omega ,\;P=\frac{\partial \Psi_{\text{mech}}}{\partial F}-pJF^{-T},\;J_{w}\cdot \nabla \mu \leq 0,\;$$

From the mixing energy (3), (9)${}_{1}$, it follows the constitutive law for the chemical potential:

(10) $$\mu_{w}=RT\left(\ln\left(1-\phi_{s}\right)+\phi_{s}+\chi \phi_{s}^{2}\right)$$

which can be rewritten, using the relation $\phi_{s}=1/\left(1+c_{d}\Omega \right)$, as:

(11) $$\mu_{w}=RT\left[\ln\left(\frac{c_{d}\Omega }{1+c_{d}\Omega }\right)+\frac{1}{1+c_{d}\Omega }+\chi {\left(\frac{1}{1+c_{d}\Omega }\right)}^{2}\right]$$

From the elastic energy (5), (9)${}_{2}$, it follows the constitutive law for the first Piola Kirchoff tensor:

(12) $$P=-pF^{-T}+G_{0}\left(F-F^{-T}\right)+2G_{0}\gamma \left(I_{4}-1\right)F\left(a_{0}⊗a_{0}\right)$$

Finally, to satisfy the remaining inequality (9)${}_{3}$, we assume that the flux $J_{w}$ is given by Chester and Anand (2010):

(13) $$J_{w}=-M\nabla \mu_{w}$$

where M is a positive definite tensor called mobility which can be related to the diffusion coefficient of water; [M]=mol${}^{2}$/(m J s).

### 2.3 Balance laws

*Mechanical equilibrium*. During cooking, the slow deformation rates typical of food processing justify neglecting inertial forces. Under this quasi-static assumption and in the absence of external body forces, the mechanical equilibrium in the current configuration reduces to:

(14) $$\nabla_{x}\cdot \sigma =0$$

In the reference frame, using the first Piola–Kirchhoff stress:

(15) ∇⋅P=0

*Mass balance*. As previously mentioned, the molar concentration of water per unit reference volume, that is dry meat is denoted as $c_{d}$. The molar concentration in the current configuration is then defined as $c=c_{d}/J$. Consequently, the mass balance in the current configuration takes the form:

(16) $$\frac{Dc}{Dt}=\overset{˙}{c}+\nabla c\cdot v=-\nabla_{x}\cdot j_{w}$$

where $\frac{D}{Dt}$ is the material derivative and v is the spatial velocity defined by $v\left(x,t\right)=\overset{˙}{u}\left(X,t\right)$ with x=f(X,t). This operator captures both the local time rate of change and the convective transport due to material motion.

In the reference frame, the mass balance can be written as:

(17) $${\overset{˙}{c}}_{d}=-\nabla \cdot J_{w}$$

being $J_{w}=JF^{-1}j_{w}$. Using Eq. (13), the mass balance could be then expressed in terms of the chemical potential as:

(18) $${\overset{˙}{c}}_{d}=\nabla \cdot M\nabla \mu_{w}$$

The relation between the current flux $j_{w}$ and the chemical potential is given by:

(19) $$j_{w}=-\overset{\sim }{M}\nabla_{x}\;\mu \;\text{with}\;M=JF^{-1}\overset{\sim }{M}F^{-T}$$

In meat, the anisotropy of mobility results from the formation of drip channels oriented parallel to the muscle fibers (van der Sman, 2017), while perpendicular diffusion follows an isotropic model. Denoting the diffusion along the fiber direction as $D_{∥}$ and $D_{⊥}$ the diffusion in perpendicular direction, after some straightforward linear algebra, the mobility in the current configuration can be written as:

(20) $$\overset{\sim }{M}=\frac{1}{RT}\left(D_{∥}\left(a⊗a\right)+D_{⊥}\left(I-\left(a⊗a\right)\right)\right)=\frac{1}{RT}\left(D_{⊥}I+D_{\Delta }\left(a⊗a\right)\right)$$

with $D_{\Delta }=D_{∥}-D_{⊥}$ the anisotropy parameter and $a=Fa_{0}/\|Fa_{0}\|$ the current director of the fiber $a_{0}$.

From (19)${}_{2}$ it follows the representation for the reference mobility M:

(21) $$M=\frac{1}{R\;T}\left(D_{⊥}C^{-1}+\frac{D_{\Delta }}{{\left\|Fa_{0}\right\|}^{2}}\;a_{0}⊗a_{0}\right)$$

*Energy balance*. In the current (deformed) configuration, the temperature evolution in meat is governed by conductive heat transfer through the tissue and convective energy transport from water migration. The energy balance simplifies to:

(22) $$\overset{˙}{e}=-\nabla_{x}\cdot q$$

where $e=\rho_{\text{eff}}c_{\text{p,eff}}T$ is the thermal energy density, with T the temperature. The effective density:

(23) $$\rho_{\text{eff}}=\phi_{s}{\overset{¯}{\rho }}_{s}+\left(1-\phi_{s}\right){\overset{¯}{\rho }}_{w}$$

where ${\overset{¯}{\rho }}_{s}$ and ${\overset{¯}{\rho }}_{w}$ are the intrinsic densities of protein and water, respectively. The effective specific heat:

(24) $$c_{p,\text{eff}}=x_{s}c_{p,s}+\left(1-x_{s}\right)c_{p,w}$$

where $x_{s}$ is the solid phase mass fraction and $x_{w}=\left(1-x_{s}\right)$ represents the water mass fraction.

The heat flux q comprises both diffusive and convective components, expressed as $q=q_{\text{diff}}+q_{\text{conv}}$ (van der Sman and Teresi, 2025). The diffusive part arises from thermal conduction and obeys Fourier’s law:

(25) $$q_{\text{diff}}=-k\nabla_{x}T$$

The thermal conductivity of meat can be modeled as direction-dependent, where the conductivity tensor k depends on the fiber orientation n in the current configuration. It is expressed as:

(26) $$k=k_{∥}\left(a⊗a\right)+k_{⊥}\left(I-\left(a⊗a\right)\right)=k_{⊥}I+k_{\Delta }\left(a⊗a\right)$$

where $k_{\Delta }=k_{∥}-k_{⊥}$. The parallel ($k_{∥}$) and perpendicular ($k_{⊥}$) conductivities are given by the volume-fraction-weighted averages of the protein and water phases (van der Sman, 2008):

(27) $$k_{∥}=\phi_{w}k_{w}+\phi_{s}k_{s},\;k_{⊥}^{-1}=\phi_{w}k_{w}^{-1}+\phi_{s}k_{s}^{-1}.$$

To map this anisotropic conductivity to the material (reference) frame, a pullback transformation is applied, consistent with standard tensor transformations in continuum mechanics:

(28) $$K=JF^{-1}kF^{-T}$$

The convective heat flux $q_{\text{conv}}$ arises from the diffusive mass flux $j_{w}$ transporting enthalpy away from the co-moving control volume, it is given by:

(29) $$q_{\text{conv}}=\rho_{w}c_{p,w}\Omega j_{w}T$$

Finally, the energy balance in the reference frame can be expressed as:

(30) $$\frac{\partial }{\partial t}\left(J\rho_{\text{eff}}c_{p,\text{eff}}T\right)=-\nabla \cdot Q$$

with:

(31) $$Q=K\nabla T-\rho_{w}c_{p,w}\Omega J_{w}T$$

### 2.4 Initial and boundary conditions

Given the first derivatives of the strain energy function (as defined in Eq. (5)) with respect to the first invariant of the right Cauchy–Green tensor $I_{1}$, denoted $\Psi_{\text{mech},1}$, and the pseudo-invariant $I_{4}$, denoted $\Psi_{\text{mech},4}$, and assuming fiber alignment along the direction $a={\overset{ˆ}{e}}_{x}$, the principal components of the Cauchy stress tensor in the initial (homogeneous, free-swollen) state reduce under transverse isotropy to:

$$\sigma_{xx}=-p+\frac{2\Psi_{\text{mech},1}}{J_{0}}\lambda_{x,0}^{2}+\frac{2\Psi_{\text{mech},4}}{J_{0}}=0$$

(32) $$\sigma_{yy}=\sigma_{zz}=-p+\frac{2\Psi_{\text{mech},1}}{J_{0}}\lambda_{z,0}^{2}=0$$

as indicated in Eq. (32), all principal components of the Cauchy stress tensor must vanish in this reference configuration.

The dry state serves as the reference configuration, with the initial swelling ratio $J_{0}=\lambda_{x,0}\lambda_{z,0}^{2}$ being equal to the inverse of the initial polymer volume fraction ($J_{0}=1/\phi_{s,0}$). The hydrostatic pressure p is determined by the chemical equilibrium:

(33) $$\mu_{w,0}=\mu_{w,mix}\left(\phi_{s,0}\right)+\Omega \;p=\mu_{w,ext}=0$$

The free swollen state is in equilibrium with pure water, which is determined by $\mu_{w,ext}=0$. $\mu_{w,mix}\left(\phi_{s,0}\right)$ is the mixing contribution to the chemical potential of water in the muscle, which follows the Flory–Huggins theory:

(34) $$\frac{\mu_{w,mix}}{RT_{0}}=\log\left(1-\phi_{s,0}\right)+\phi_{s,0}+\chi \left(T_{0}\right)\phi_{s,0}^{2}$$

Thus, for any given transversely isotropic strain energy functional expressed in terms of $I_{1}$ and $I_{4}$, the initial state can be computed accordingly. In Fig. 1 the initial free-swollen state of a meat sample with dimensions $L_{d}\times L_{d}\times e$ and fiber orientation defined by $a_{0}$ is illustrated. To find the free- swollen state B, the chemical equilibrium condition (Eq. (33)) is applied, resulting in two principal stretches: one along the fiber direction, $\lambda_{∥,0}$, and one transverse to it, $\lambda_{⊥,0}$, both of which are greater than one and providing a modified meat geometry of $\lambda_{∥,0}L_{d}\times \lambda_{⊥,0}L_{d}\times \lambda_{⊥,0}e$. If a reference volume element (RVE) of size $L_{s}\times L_{s}\times e^{\prime }$ is selected, then upon applying a uniform temperature increase ΔT>0, the RVE will progressively dry, resulting in actual dimensions $\lambda_{∥}L_{s}\times \lambda_{⊥}L_{s}\times \lambda_{⊥}e^{\prime }$, where $\lambda_{∥}<1$ and $\lambda_{⊥}<1$. Consequently, the muscle fiber direction vector will shorten accordingly, becoming $a=\lambda_{∥}\left(\lambda_{∥,0}a_{0}\right)$.

In this work, the initial principal stretches that define the initial swollen, stress-free state are obtained through a stationary analysis in COMSOL Multiphysics, implementing the aforementioned equations.

Following van der Sman and Teresi (2025), the mass flux due to evaporation at the free boundaries of the meat is given by:

(35) $$j_{w,ext}\cdot n=-M\nabla \mu_{w}\cdot n=\beta \left(c_{v,\text{surf}}-c_{v,ext}\right)$$

in this expression, n is the normal unit vector of the surface in the current frame, $c_{v,ext}$ is the vapour concentration in the air, and β is the mass transfer coefficient. $c_{v,\text{surf}}$ is the equilibrium vapour concentration at the surface, determined by the liquid’s saturation properties.

(36) $$c_{v,\text{surf}}=a_{w}c_{\text{sat}}\left(T\right)$$

where $a_{w}$ is the water activity and is related to the chemical potential $\mu_{w}=RT\log\left(a_{w}\right)$. The saturated vapour concentration $c_{\text{sat}}$ is related to the saturated vapour pressure:

(37) $$c_{\text{sat}}\left(T\right)=\frac{p_{\text{sat}}\left(T\right)}{RT}$$

$p_{sat}$ is temperature dependent, following well-known relations like the Clausius-Clapeyron relation.

$c_{v,\text{ext}}$ is often specified via the relative humidity $RH_{\text{air}}$ (van der Sman and Teresi, 2025):

(38) $$c_{v,\text{ext}}=RH_{\text{air}}c_{\text{sat}}\left(T_{\text{air}}\right)$$

The current model assumes evaporation occurs only at the surface. At temperatures exceeding 100 °C, corresponding to roasting conditions, the formation of a glassy crust may occur, leading to internal evaporation driven by boiling and the development of porosity. These phenomena will be the subject of future investigation.

Beyond evaporation, the cooking simulations account for liquid water migration through the specified drip channels, resulting in drip loss at the boundaries. The drip flux is considered as:

(39) $$j_{w,drip}\cdot n=\left(\phi_{w}-\phi^{eq}\right)\delta$$

where the difference $\left(\phi_{w}-\phi^{eq}\right)$ can be interpreted as the excess water that drips out, where $\phi^{eq}$ represents the water holding capacity (Moya et al., 2021), which is temperature dependent. The parameter δ characterizes the rate of water dripping. Note, that dripping does not involves extraction of latent heat from the product, as evaporation is not involved. The dripped juice might evaporate if in contact with the frying pan, but this will not extract energy from the meat.

The heat and mass transfer at the boundary are coupled to each other due to the evaporative cooling effect, which is expressed as:

(40) $$q_{\text{ext}}=h\left(T_{\text{ext}}-T\right)+j_{w,\text{ext}}\cdot n\Omega \left(\Delta H_{\text{evap}}+c_{p,v}T\right)+j_{w,drip}\cdot n\Omega \left(c_{p,v}T\right)$$

where $\Delta H_{\text{evap}}$ is the (molar) latent heat of evaporation. The relation between heat and mass transfer coefficient is the Lewis relation (van der Sman and Teresi, 2025):

(41) $$\beta =\frac{h}{\rho_{\text{air}}c_{p,\text{air}}}$$

$c_{p,\text{air}}$ is the specific heat of air and $\rho_{\text{air}}$ is the mass density of air.

During cooking, the contact between the meat and the pan is modeled as a heat transfer interaction given by:

(42) $$q_{\text{frying}}=h_{c}\left(T_{\text{pan}}-T\right)+j_{w,\text{ext}}\cdot n\Omega \left(\Delta H_{\text{evap}}+c_{p,v}T\right)+j_{w,drip}\cdot n\Omega \left(c_{p,v}T\right)$$

where $T_{\text{pan}}$ is the temperature at the surface of the pan. The first term models conductive heat transfer driven by the temperature difference between the pan surface and the food. The second term accounts for energy loss due to water vapour leaving the food surface, including both the latent heat of evaporation and the sensible heat carried by the vapour at the food temperature. The third term represents the sensible heat carried away by liquid water dripping from the food. Together, these terms capture the combined effects of conduction, evaporation, and moisture removal on the heat transfer during frying.

### 2.5 Computational model

**Fig. 2:**
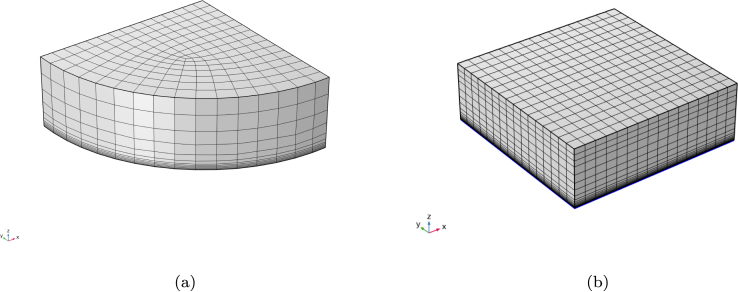
(a) Finite element mesh of the symmetric section of the domain used to simulate burger cooking. (b) Mesh of the symmetric portion of the steak model.

The equations presented in the previous section have been implemented in the finite element software COMSOL Multiphysics. Two geometries were considered in the simulations (Fig. 2), each representing a quarter of the full domain by exploiting symmetry. The first geometry corresponds to a quarter-cylinder (100 mm diameter × 20 mm height), representing a hamburger patty, and assumes isotropic material properties since ground meat lacks directional structure. The second geometry is a quarter rectangular parallelepiped (60 mm width × 60 mm depth × 20 mm height), representing an idealized steak with anisotropic properties. For the steak model, different fiber orientations were explored to test the mathematical model.

Due to the high gradients of temperature, water concentration, and deformation that occur during contact cooking with the pan, a boundary layer mesh was employed near the heated surface to accurately capture these variations. All balance equations employ quadratic Lagrangian elements, while the incompressibility condition uses linear discontinuous Lagrangian elements. For numerical stability, the pressure field elements are maintained one order lower than those of the deformation field (van der Sman and Teresi, 2025).

## 3 Results

The principal parameters employed in the different models to generate the subsequent numerical results are systematically compiled in Table 1. It includes material properties such as the shear modulus and fiber reinforcement factor, as well as parameters for the Flory–Huggins interaction model. Water diffusion properties are also listed, including anisotropic diffusivity along fibers, water holding capacity obtained from Moya et al. (2021), and a scaling factor for diffusivity. Finally, the table presents heat transfer parameters, specifying convection coefficients for air and pan contact, along with thermal conductivities for water and protein components, reflecting the anisotropic nature of heat conduction in the material.

**Table 1:** Model input parameters.

Name and description	Value
**Material parameters**(Liu et al., 2015)	
Shear modulus $G_{0}$	100 kPa
Fiber reinforcement factor γ	2

**Flory–Huggins interaction parameter**(van der Sman, 2013)	
$\chi_{0}$	0.5
$\chi_{1}=\chi_{0}+\frac{\chi_{0}-\chi_{\infty }}{1+a\exp\left(-b\left(T-T_{d}\right)\right)}$	
$\chi_{1,0}$	0.8
$\chi_{1,\infty }$	1.55
a	30
b	−0.25 K^−1^
Denaturation temperature $T_{d}$	325 K

**Water diffusion**	
Along fibers diffusivity $D_{\left|\right|}=\left\{\begin{array}{cc} f_{D}\left(\phi_{w}-\phi^{eq}\right)D_{⊥}\; & \phi_{w}>\phi^{eq}\\ 0\; & \text{otherwise} \end{array}\right)$	
Water holding capacity $\phi^{eq}$ (Moya et al., 2021)	$2.986-\frac{1.69}{1+0.56\exp-0.83\left(T-66.76\right)}$$\frac{\text{kg water}}{\text{kg dry material}}$
$f_{D}$ fitted	100
$D_{⊥}$ fitted	$1\cdot 10^{-5}$ m${}^{2}$/s

**Heat transfer**(Hernández-Alhambra et al., 2024)	
Air convection coefficient h	8 W/(m${}^{2}\;$K)
Pan contact heat transfer coefficient $h_{c}$	200 W/(m${}^{2}\;$K)
Water heat conductivity $k_{\left|\right|}$	0.6 W/(m K)
Protein heat conductivity $k_{⊥}$	0.2 W/(m K)
Pan surface temperature $T_{\text{pan}}$	140 °C

**Fig. 3:**
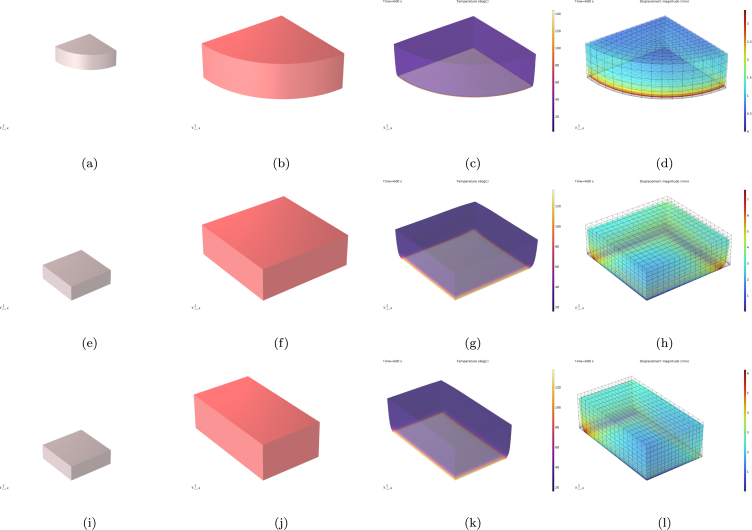
Burger model: (a) Dry state, (b) free swollen state, (c) temperature distribution after 600 s and (d) displacement magnitude field after 600 s superimposed with the finite element mesh at the free swollen state. Isotropic steak model: (e) Dry state, (f) free swollen state, (g) temperature distribution after 600 s and (h) displacement magnitude field after 600 s superimposed with the finite element mesh at the free swollen state. Anisotropic steak model with fibers parallel to the x-axis: (i) Dry state, (j) free swollen state, (k) temperature distribution after 600 s and (l) displacement magnitude field after 600 s superimposed with the finite element mesh at the free swollen state.

Fig. 3 presents the thermal and mechanical response of both food models after 600 s of cooking, including the same steak model treated as isotropic for comparative purposes. Fig. 3, Fig. 3, Fig. 3 show the dry state considered for the simulations, whereas Fig. 3, Fig. 3, Fig. 3 correspond to the stress-free swollen state at the beginning of the cooking simulations. Fig. 3, Fig. 3 present the temperature distributions for the hamburger patty and isotropic steak, while Fig. 3(k) shows the temperature distribution for the steak considered as anisotropic. Panels 3(d), Fig. 3, Fig. 3 display the corresponding displacement magnitude fields, illustrating the meat’s retraction during cooking with the finite element mesh superimposed at the initial swollen state. The steak model incorporates muscle fibers aligned parallel to the x-axis, demonstrating anisotropic behavior.

**Fig. 4:**
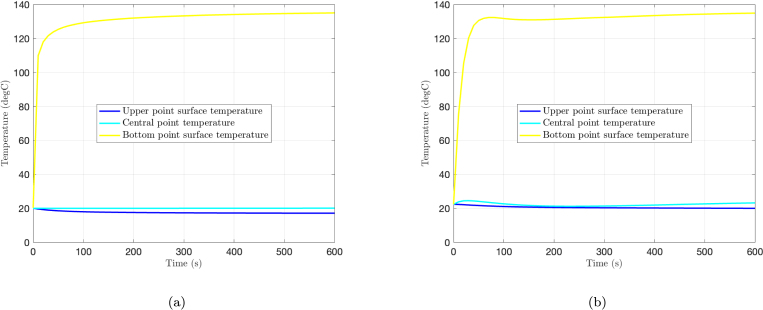
Temperature evolution during cooking at three points across the thickness of (a) the hamburger patty model and (b) the meat steak model.

The temperature evolution during cooking at three point locations across the thickness is shown in Fig. 4 for both the hamburger patty and the anisotropic meat steak models. The results for the isotropic steak have been omitted since are equivalent to those of the hamburger. Surface points heat up rapidly due to direct contact with the pan, while internal regions show a slower, diffusive increase in temperature. These thermal profiles are in good agreement with previous experimental observations reported in Hernández-Alhambra et al. (2024) for hamburger patties and in Moya et al. (2021) for meat steaks, validating the model’s ability to capture the characteristic temperature gradients that develop during pan frying.

**Fig. 5:**
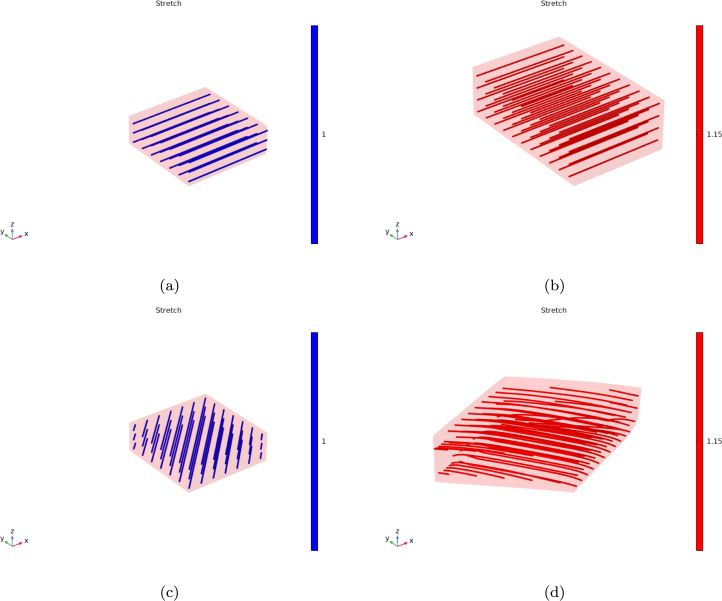
Unswollen and swollen configurations of the idealized steak showing fiber orientation and stretch: (a, b) fibers parallel to the x-axis. (c, d) fibers at 45°to the x-axis.

An example of how the swollen configuration is obtained prior to initiating the cooking simulation is shown in Fig. 5, which compares the unswollen and swollen configurations of the idealized steak geometry. In both cases, the fiber orientation and along fiber stretch are visualized. Fig. 5, Fig. 5 correspond to fibers initially aligned with the x-axis, while Fig. 5, Fig. 5 illustrate fibers oriented at 45°to the x-axis. The swelling-induced deformation generates both axial (along-fiber) and transverse (cross-fiber) stretching, establishing a free-swollen initial state where mechanical stresses balance the hydrostatic pressure from the chemical potential.

**Fig. 6:**
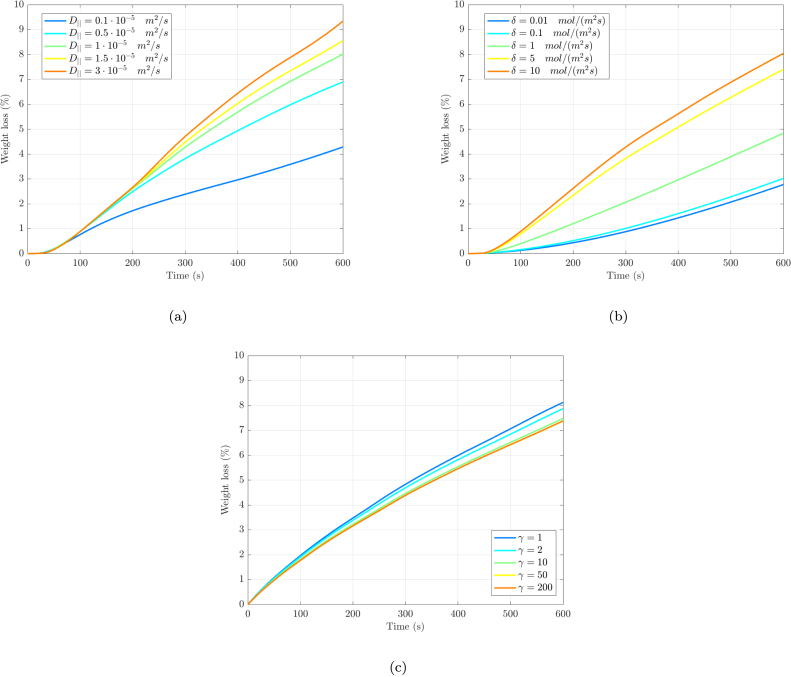
(a) Influence of diffusion along fibers ($D_{∥}$) on total weight loss in the idealized steak geometry. (b) Impact of drip rate coefficient (δ) on total weight loss in the same geometry. (c) Effect of fiber reinforcement (γ) on total weight loss in the idealized steak geometry.

In Fig. 6 the effect of key parameters governing fluid transport in cooked steak is presented. Fig. 6(a) shows that increasing the along-fiber diffusion coefficient ($D_{∥}$) significantly increases total weight loss. Enhanced parallel diffusion facilitates greater water transport to the boundary, where both surface evaporation and dripping mechanisms are amplified. Fig. 6(b) shows a nonlinear dependence of weight loss on the drip rate coefficient (δ) for a given diffusion coefficient ($D_{∥}=1\cdot 10^{-5}$ m${}^{2}$/s in this case). Below 0.1 mol/(m${}^{2}$ s), δ variations have negligible effect. Similarly, at high δ values, weight loss remains nearly the same as the system reaches maximum exudation capacity since no additional water remains available for drainage. This saturation behavior demonstrates how cooking losses are ultimately constrained by both boundary conditions (water availability) and internal transport (governed by $D_{∥}$). Finally, as shown in Fig. 6(c), the anisotropy of the meat, governed by the fiber reinforcement parameter (γ), also influences water loss, and as presented, increased stiffness tends to reduce water loss. However, this effect is notably weaker than that of the previously discussed parameters.

**Fig. 7:**
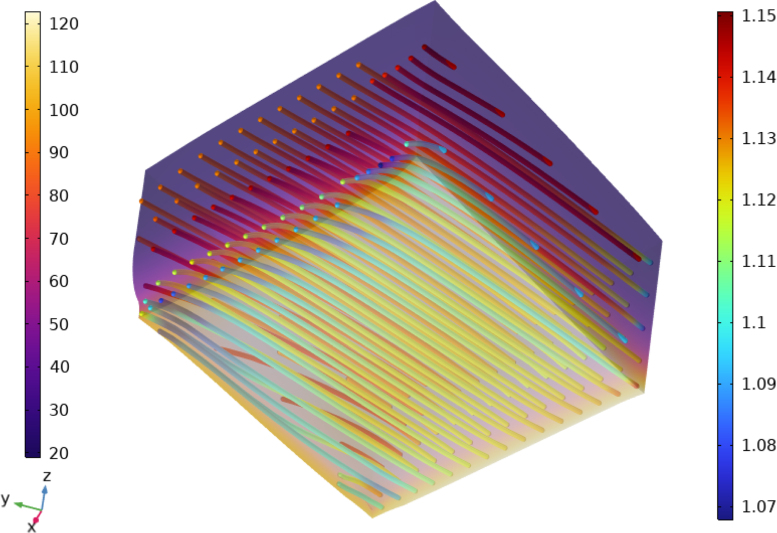
Detail of the steak model’s bottom surface at t=600 s with initial fiber orientation at 45°with respect to x-axis. Temperature distribution in degC (left color legend) with a transparency filter and along-fiber stretch distribution (right color legend) across the discretized fiber network. (For interpretation of the references to color in this figure legend, the reader is referred to the web version of this article.)

A detailed view of the steak model’s bottom surface showing the deformed configuration and fiber stretch at the end of the pan frying simulation (t=600 s) is presented in Fig. 7. The visualization superimposes the temperature distribution (in °C), revealing how the temperature gradient influences fiber shortening. Heated regions exhibit greater along-fiber deformation.

## 4 Conclusions

This paper presents a multiphysics model developed to simulate the large deformation behavior of meat-like materials during pan frying, with a particular focus on fiber stretch, swelling, and heat transfer. The model accounts for the coupled effects of thermal conduction, moisture-induced swelling, and anisotropic elasticity to capture the complex morphological changes observed in realistic food geometries. We study two idealized configurations: a cylindrical hamburger patty and a steak-like slab with embedded fiber orientation. Prior to cooking, the hydrated (swollen) state is computed, serving as the initial condition for the frying simulation. The temperature evolution across the sample thickness matches experimental trends reported in Hernández-Alhambra et al. (2024) for hamburger patties and in Moya et al. (2021) for steaks. Deformation is strongly influenced by fiber orientation and temperature gradients, with greater along-fiber shortening observed in heated regions. This framework provides a predictive tool for understanding and designing cooking processes of structured food materials and may also serve as a foundation for simulating shape morphing in thermally actuated edible systems or synthetic thermoresponsive materials.

## Declaration of competing interest

The authors declare the following financial interests/personal relationships which may be considered as potential competing interests: Jorge Grasa reports financial support was provided by Spanish Ministry of Science and Innovation. Jorge Grasa reports financial support was provided by European Union. Jorge Grasa reports financial support was provided by Government of Aragón. Jorge Grasa reports a relationship with BSH Home Appliances Group that includes: funding grants. If there are other authors, they declare that they have no known competing financial interests or personal relationships that could have appeared to influence the work reported in this paper.
